# Analysis of thermomechanical stresses in dual compound thick cylinders under asymmetric loads: An analytical and numerical method

**DOI:** 10.1016/j.heliyon.2024.e24938

**Published:** 2024-01-23

**Authors:** Palash Das, Md Ashraful Islam, Dipayan Mondal

**Affiliations:** a Department of Mechanical Engineering, Khulna University of Engineering & Technology, Khulna -9203, Bangladesh; b Department of Mechanical Engineering, Bangladesh Army University of Science and Technology, Saidpur, Bangladesh

**Keywords:** Compound cylinder, Asymmetric loading, Finite difference method, Thermoelastic analysis

## Abstract

This study presents a 2D comprehensive analytical and numerical analysis of the thermomechanical stresses in an unsymmetric dual compound thick cylinder under steady-state conditions. By employing mathematical analysis, this research aims to investigate the effectiveness of a 2D compound cylinder in reducing elastic and thermoelastic stresses. The temperature and displacement fields are thought to be dependent on the radial and circumferential directions, subject to asymmetric thermal and mechanical boundary conditions on the inner and outer surfaces. In this scenario, the Poisson ratio is considered to be a constant. The techniques of variable separation and complex Fourier series are employed analytically in the solution of heat conduction and Navier equations. The results obtained from the developed analytical method are compared and validated against those obtained from a finite difference method (FDM). The findings of this study suggest that the clamping of the outer layer has a significant influence on stress distribution in the structure, and the impact of tangential stress on the behavior of a compound cylinder is highly dominant. Furthermore, changes in temperature significantly influence hoop stress compared to variations in internal pressure levels. Moreover, the influence of internal pressure is relatively attenuated when a pressure vessel is fabricated utilizing different metals. In addition, the findings indicated that the configuration of layers and the location of the highest temperature had a significant impact on the performance of the vessel. Nevertheless, the technology provided has sufficient robustness to effectively address the complexities associated with the design of multilayered graded materials (GM) in additive manufacturing applications.

## Nomenclature

*i* = 1,2: Inner cylinder and outer cylinder
*a, b, c*: Compound cylinder's radius
*T*^*i = 1,2*^: Temperature distribution on inner and outer cylinder respectively
*r*: Radial coordinate
θ: Tangential coordinate
$C_{ij}$: Conduction and convection parameters
$f_{i}$: Convection functions
$A_{ni}^{i}$: Integration constants
$k^{i}$: Thermal conductivities

**$\xi^{i},E_{ij}$ Parameters**
*q*: Heat flux
$u^{i}$: Radial displacement function
$v^{i}$: Tangential displacement function
$\varepsilon_{ij}^{i}$: Strain components
$\sigma_{rr}^{i}$: Radial stress components
$\sigma_{\theta \theta }^{i}$: Hoop stress components
$\sigma_{r\theta }^{i}$: Shear stress components
$\lambda^{i},\mu^{i}$: Lame's constant
$\nu^{i}$: Poisson's ratio
$\alpha^{i}$: Thermal expansion coefficient
$\Phi_{i}$: Known function
$T_{m,n}^{i}$: Temperature in *i* = 1,2 cylinder in *m, n* nodes
*m, n*: Nodal points along the radial and tangential direction respectively
$P_{m,n}^{i}$: Pressure on inside and outside nodal points

## 1 Introduction

Cylinders are structural elements that possess the ability to confine fluids at elevated pressure and temperature. They are frequently employed in gas and high-pressure vessels, chemical and nuclear facilities, and the oil and gas industry [1]. However, cylinders can be categorized into two distinct types: thin-walled cylinders and thick-walled cylinders. According to the traditional Lame's theory of thick-walled cylinders [2], the cylinder will burst if the internal fluid pressure gets too close to the maximum allowable safe operating stress for the material. To tackle these challenges effectively, it is recommended to employ compound cylinders.

Compound cylinders consist of two or more individual cylinders. The fabrication of compound cylinders typically involves several manufacturing processes, including hydraulic expansion, thermo-hydraulic expansion, and forming welded and riveted plates. Alternatively, the cylinders can be narrowed by inserting one cylinder into another with varying levels of diametrical interference [3]. The occurrence of shrinkage can lead to the formation of residual stress distribution within the walls of a cylinder, consequently augmenting their capacity to endure elevated levels of pressure compared to a single cylinder.

However, the presence of axisymmetric or asymmetric thermomechanical load on cylindrical or spherical structures is inevitable in their practical applications. A lot of research has been conducted so far under these circumstances. Arefi et al. [4] investigated reinforced graphene nanoplate using shear deformation and potential energy theory subjected to thermo-mechanical loads. The study demonstrates that the number of layers substantially impacts the distribution of failure stress. According to their report, it was found that stress levels decrease with an increase in layers. Zhao et al. [5] present a study investigating the snap-through buckling phenomenon in a spherical shell subjected to combined thermal and mechanical loads. The findings clarify that as the radius of the bottom circle expands, there is a decrease in the lower critical load of shells and an increase in the jump amplitude of buckling. Additionally, the authors conclude that the upper and lower critical loads will grow as shell thickness grows. Another investigation of a rotating functionally graded material (FGM) cylinder subjected to the aforementioned loading has been conducted [[6], [7], [8]]. This study examines the impact of the nonhomoginity parameter on design considerations. Benslimane et al. [9] have done magneto-mechanical analysis along with the thermo-mechanical environment. The findings reveal that the Lorentz force has a substantial influence when designing a rotating cylindrical vessel in a magnetic environment. A hyperplastic cylinder's torsional and swelling characteristics have been investigated [10]. According to the study, it was found that when a larger torsional twist is applied to the structure, it creates greater stress components and generates a larger radial deformation.

The paper by Bai et al. [11] presents a novel model that accounts for the coupled thermo-hydro-mechanical process in soils. This model departs from conventional conceptions and incorporates the influences of loading routes and soil structure. However, Zhu et al. [12] devised the ameliorated longitudinal critically refracted-attenuation velocity method, a novel, and enhanced method, for determining residual stress in welding. In order to reduce the low-frequency vibration of high-precision instruments, Rong-Biao Hao et al. [13] investigated an orthogonal six-DOFs vibration isolation system with configurable high-static-low-dynamic stiffness properties. Jun Wang et al. [14] have developed a parallel robot equipped with a constant force actuator specifically designed for grinding applications. The research involves developing solutions for determining the robot's spatial positions and poses, along with analyzing the forces generated by the system. Moreover, the collapse resistance of steel frames has been studied by Li-min Tian et al. [15]. It showed that the improvement was caused by including innovative connections that utilize corrugated steel plates welded between the inner flange of I-shaped beams and the column. Jiahao Shi et al. [16] analyzed thermal electrohydrodynamic (TEHD) lubrication of a journal-thrust coupled bearing in diesel engines at high loads and temperatures. Parametric analyses investigate the combined effects of temperature and pressure on lubrication performance and dynamic properties. The interfacial characterization of the butter joint of hot-rolling CP-Ti/Q235 bimetallic sheets was examined by Zhur et al. [17] using Laser + CMT. This study investigates the impact of heat input on the creation of intermetallic compounds (ICs) in composite structures composed of titanium and carbon steel. In their study, Cao et al. [18] examined the production and attributes of PLA and PLA/CF samples using the FFF method. They showed valuable insight into these materials' thermal stability and mechanical characteristics, which might be useful for a wide range of industries. A further investigation demonstrated the influence of temperature-related fit clearance on the sound radiation capabilities of Full Ceramic Ball Bearings (FCBB) [19]. The findings revealed a notable impact of fit clearance on the emission of sound as a result of modified interactions among FCBB components. In their study, Mengyu Cao et al. [20] investigate the existence of subsurface mesoscale fractures in the outer ring of Full Ceramic Ball Bearings (FCBBs). The paper presents a dynamic model, utilizing the strain energy theory to examine the influence of varying fracture lengths on the operational condition. Another research based on gaseous hydrogen fuel has been studied by Zhilong Cheng et al. [21]. This research looks at the effect of methane and oxygen injection concentrations on sintering performance in sinter plants, with the goal of partially replacing solid fossil fuel with hydrogen-based gaseous fuel.

Furthermore, the effects of temperature on a fluid-filled FGM cylinder were investigated using higher-order shear deformation theory and the Pasternak elastic foundation [22]. Along with the elastic analysis, elastoplastic analysis has been done on rotating FGM cylinders [[23], [24], [25], [26]]. The studies conducted by Arslan et al. and Sim et al. [27,28] are reported to investigate the application of a functionally graded material (FGM) composed of steel and aluminum in a spherical container under combined thermo-mechanical loading conditions. The findings reveal that the utilization of FGM comprising both aluminum and steel can result in a reduction of approximately 50 % in weight compared to the individual use of either aluminum or steel. In addition to ambient temperature cooling, cryogenic cooling has also been documented as a method that involves the application of sudden high-temperature gradients, leading to the generation of induced high thermal stress [29].

Numerous research studies have been undertaken and documented in the scientific literature pertaining to compound cylinders. Parker [30] extended the variable material properties (VMP) method to examine shrink-fit and autofrettage's impact on compound cylinders. Majzoobi et al. [31] found that the best shrinking radius of compound cylinders exposed to bursting and autofrettage pressure could be achieved when the outer-to-inner cylinder radii ratio equals 2.2. According to Parker and Kendall [32], a proposed manufacturing process involves shrink-fitting two tubes before autofrettage to optimize the tubes' fatigue life. When calculating the stress intensity factor in the compound cylinder, they considered the various yielding scenarios that may have occurred at the inner and outer radii of the liner, as well as the yielding that could have occurred at the outer radius of the jacket. Jahed et al. [33,34] introduced an optimization approach to VMP to identify the optimal design of a three-layered vessel for maximum fatigue life expectancy under the combined impacts of autofrettage and shrink-fit. This was done to maximize the capacity of the vessel to withstand fatigue damage. The VMP approach was utilized by Mohammadi et al. [35] in order to investigate the residual stresses that were caused by compound cylinders of different geometries.

Aside from the aforementioned thermomechanically loaded structures, a few works exist on asymmetric cylindrical pressure vessels. The problem of analyzing an asymmetric cylindrical pressure vessel subjected to combined thermal and mechanical loading has been addressed using complex Fourier series and other analytic methods [[36], [37], [38]]. The researchers employ an analytical method to solve the heat equation and the equation of motion in the presence of a steady load. Additionally, it has been demonstrated that considering asymmetric load is crucial from a design perspective, as it can generate shear load that ultimately leads to structural failure.

Advancements in compound or sandwich structures have been a focal point in the 21st century, with significant efforts being made to enhance their performance under various loads. Given the foregoing, multilayered or compound structures may have an opportunity to compete against their contemporaries. However, the novel idea that arises from creating practical FGM material [39] encounters challenges in accurately ensuring gradual volume fraction variation. As a result, we have been drawn to layer-wise models or compound structures. There appears to be a lack of study, namely by K. Bahoum [1], in the area of revealing the performance of compound cylinders subjected to symmetric thermo-mechanical loading. To the best of the authors’ knowledge, there is limited analytical and numerical documentation on the behavior of metallic compound cylinders subjected to asymmetric loads. In this particular study, the authors aim to analytically and numerically expand a one-dimensional (1D) axisymmetric physical problem to a two-dimensional (2D) non-axisymmetric problem, investigated by K. Bahoum [1]. In order to obtain an analytical solution, the energy equation and Navier equation were solved utilizing complex Fourier series. On the other hand, the numerical solution was obtained using the finite difference method (FDM).

## 2 Heat conduction problem

**Fig. 1:**
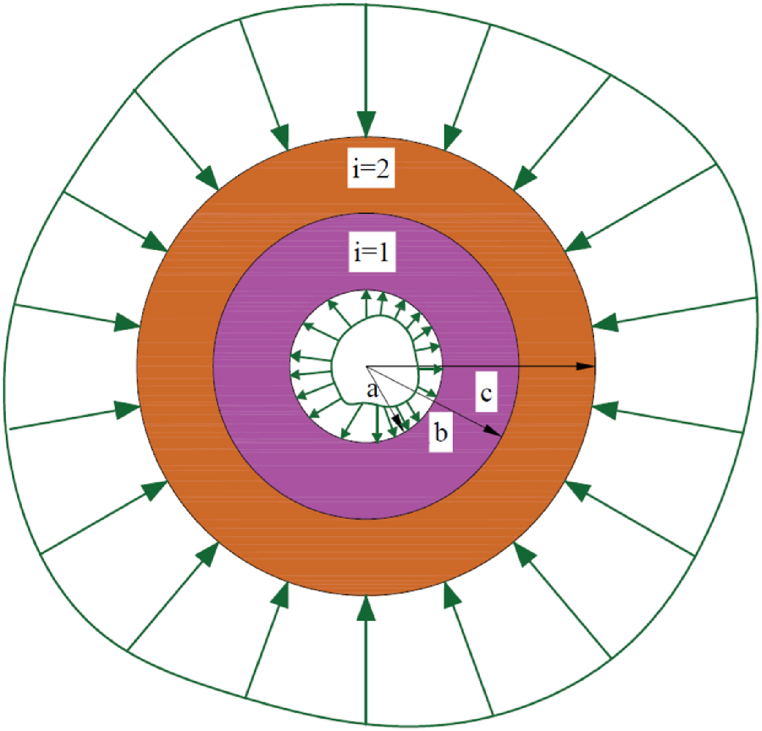
Illustration of asymmetrically loading multilayered cylinder.

A compound cylinder with the radii a, b, and c, respectively, composed of two isotropic homogeneous materials is being studied under steady state conditions where no heat is being generated. The schematization of the model is shown in Fig. 1.

The heat conduction equation for these types of hollow cylinders is given as [40]:

(1) $$\frac{\partial^{2}T^{i}\left(r,\theta \right)}{\partial r^{2}}+\frac{1}{r}\frac{\partial T^{i}\left(r,\theta \right)}{\partial r}+\frac{1}{r^{2}}\frac{\partial^{2}T^{i}\left(r,\theta \right)}{\partial \theta^{2}}=0\;\text{Where}\;i=1,2$$

It is subjected to the general linear boundary conditions that are

(2) $$\begin{array}{l} C_{11}T^{i}\left(a,\theta \right)+C_{12}\frac{\partial T^{i}\left(a,\theta \right)}{\partial r}=f_{1}\left(\theta \right)\\ C_{21}T^{i}\left(c,\theta \right)+C_{22}\frac{\partial T^{i}\left(c,\theta \right)}{\partial r}=f_{2}\left(\theta \right) \end{array}$$

where $C_{ij}$ are thermal conduction and convection parameters. $f_{1}\left(\theta \right),f_{2}\left(\theta \right)$ are known parameters on the inner and outer radii, respectively. Because the compound cylinder in this research is subjected to periodic boundary conditions (Table 4), the solution may be represented as a complex Fourier series as:

(3) $$T^{i}\left(r,\theta \right)=\sum_{n=-\infty }^{\infty }T_{n}^{i}\left(r\right)e^{in\theta }$$

where $T_{n}^{i}$ is the coefficient of complex Fourier series is

(4) $$T_{n}^{i}=\frac{1}{2\pi }\int_{-\pi }^{\pi }T^{i}\left(r,\theta \right)e^{-in\theta }$$

Substituting Eq. (3) to Eq. (1), the following equation can be obtained:

(5) $$r^{2}\frac{d^{2}T_{n}^{i}}{dr^{2}}+r\frac{dT_{n}^{i}}{dr}-n^{2}T_{n}^{i}=0$$

Eq. (5) is an ordinary Cauchy-Euler differential equation. The solution of the above equation is

(6) $$\begin{array}{l} T_{n}^{i}=A_{n1}^{i}r^{\varsigma_{n1}^{i}}+A_{n2}^{i}r^{\varsigma_{n2}^{i}}\\ \text{Where},\xi^{i}=\pm n \end{array}$$

Substituting Eq. (6) into Eq. (3), gives

(7) $$T^{i}\left(r,\theta \right)=\sum_{n=-\infty }^{\infty }\left(A_{n1}^{i}r^{\varsigma_{n1}^{i}}+A_{n2}^{i}r^{\varsigma_{n2}^{i}}\right)e^{in\theta }\;\text{Where},i=\left[1,2\right]$$

The following condition is established when two homogenous materials are perfectly joined (no thermal contact resistance)

(8) $$\begin{array}{l} {T^{i}\left(b,\theta \right)|}_{i=1}={T^{i}\left(b,\theta \right)|}_{i=2}\\ {k^{i}\frac{\partial T^{i}\left(b,\theta \right)}{\partial r}|}_{i=1}={k^{i}\frac{\partial T^{i}\left(b,\theta \right)}{\partial r}|}_{i=2}\\ \frac{k^{i}}{r}{\frac{\partial T^{i}\left(b,\theta \right)}{\partial \theta }|}_{i=1}=\frac{k^{i}}{r}{\frac{\partial T^{i}\left(b,\theta \right)}{\partial \theta }|}_{i=2} \end{array}$$

The system of equations is formed by Eq. (2 a, b) and Eq. (8 a, b, c) as:

$$\begin{array}{l} \left[\begin{array}{cccc} E_{11} & E_{12} & 0 & 0\\ b^{\zeta_{n1}^{i}} & b^{\zeta_{n2}^{i}} & -b^{\zeta_{n1}^{i}} & -b^{\zeta_{n2}^{i}}\\ \zeta_{n1}b^{\zeta_{n1}^{i}-1} & \zeta_{n2}b^{\zeta_{n2}^{i}-1} & -\zeta_{n1}b^{\zeta_{n1}^{i}-1} & -\zeta_{n2}b^{\zeta_{n2}^{i}-1}\\ 0 & 0 & E_{43} & E_{44} \end{array}\right]\left\{\begin{array}{c} A_{n1}^{1}\\ A_{n2}^{1}\\ A_{n1}^{2}\\ A_{n2}^{2} \end{array}\right\}=\left\{\begin{array}{c} \frac{1}{2\pi }\int_{-\pi }^{\pi }f_{1}\left(\theta \right)d\theta \\ 0\\ 0\\ \frac{1}{2\pi }\int_{-\pi }^{\pi }f_{2}\left(\theta \right)d\theta \end{array}\right\}\\ \text{Where};\;E_{11}=C_{11}a^{\xi_{n1}^{i}}+C_{12}\xi_{n1}a^{\xi_{n1}^{i}-1}\\ E_{12}=C_{11}a^{\xi_{n2}^{i}}+C_{12}\xi_{n2}a^{\xi_{n2}^{i}-1}\\ E_{43}=C_{21}c^{\xi_{n1}^{i}}+C_{22}\xi_{n1}c^{\xi_{n1}^{i}-1}\\ E_{44}=C_{21}c^{\xi_{n2}^{i}}+C_{22}\xi_{n2}c^{\xi_{n2}^{i}-1} \end{array}$$

## 3 Stress analysis

As the cylinder's third direction is so large, the plain strain condition has been made for brevity, disregarding the influence of end effects. Let u and v the displacement components in the radial and circumferential direction, respectively.

The following are the strain-displacement relationships [41]:

(9) $$\varepsilon_{rr}=\frac{\partial u\left(r,\theta \right)}{\partial r},\varepsilon_{\theta \theta }=\frac{1}{r}\frac{\partial v\left(r,\theta \right)}{\partial \theta }+\frac{u\left(r,\theta \right)}{r},\varepsilon_{r\theta }=\frac{1}{2}\left(\frac{1}{r}\frac{\partial u\left(r,\theta \right)}{\partial \theta }+\frac{\partial v\left(r,\theta \right)}{\partial r}-\frac{v\left(r,\theta \right)}{r}\right)$$

The stress-strain relationship can be written in the elastic zone as [42]:

(10) $$\left\{\begin{array}{c} \sigma_{rr}^{i}\\ \sigma_{\theta \theta }^{i}\\ {\sigma_{r\theta }}^{i} \end{array}\right\}=\left[\begin{array}{cccc} \left(\lambda^{i}+2\mu^{i}\right) & \lambda^{i} & -\left(3\lambda^{i}+2\mu^{i}\right)\alpha^{i} & 0\\ \lambda^{i} & \left(\lambda^{i}+2\mu^{i}\right) & -\left(3\lambda^{i}+2\mu^{i}\right)\alpha^{i} & 0\\ 0 & 0 & 0 & 2\mu^{i} \end{array}\right]\left\{\begin{array}{c} \varepsilon_{rr}^{i}\\ \varepsilon_{\theta \theta }^{i}\\ T^{i}\left(r,\theta \right)\\ \varepsilon_{r\theta }^{i} \end{array}\right\}$$

where λ,μ are lame's constants [36]. $\sigma_{jk}^{i}$ and $\varepsilon_{jk}^{i}$ are stress and strain tensors.

Steady state conditions must be established without taking into account inertia and body force, from the stress tensor the equilibrium equations in the radial and circumferential directions are as follows:

(11) $$\begin{array}{l} \frac{\partial \sigma_{rr}^{i}}{\partial r}+\frac{1}{r}\frac{\partial \sigma_{r\theta }^{i}}{\partial \theta }+\frac{1}{r}\left(\sigma_{rr}^{i}-\sigma_{\theta \theta }^{i}\right)=0\\ \frac{\partial \sigma_{r\theta }^{i}}{\partial r}+\frac{1}{r}\frac{\partial \sigma_{\theta \theta }^{i}}{\partial \theta }+\frac{2}{r}\sigma_{r\theta }^{i}=0\;\text{Where}\;i=\left[1,2\right] \end{array}$$

The Navier equations in terms of displacements are calculated using the relationships Eqs. (9), (11).

(12) $$r^{2}\frac{\partial^{2}u^{i}\left(r,\theta \right)}{\partial r^{2}}+r\frac{\partial u^{i}\left(r,\theta \right)}{\partial r}-u^{i}\left(r,\theta \right)+\left(\frac{1-2\nu^{i}}{2-2\nu^{i}}\right)\frac{\partial^{2}u^{i}\left(r,\theta \right)}{\partial \theta^{2}}+\left(\frac{1}{2-2\nu^{i}}\right)r\frac{\partial^{2}v^{i}\left(r,\theta \right)}{\partial r\partial \theta }+2\left(\frac{\nu^{i}-3}{1-\nu^{i}}\right)\frac{\partial v^{i}\left(r,\theta \right)}{\partial \theta }=\left(\frac{1+\nu^{i}}{1-\nu^{i}}\right)\alpha^{i}r^{2}\frac{\partial T^{i}\left(r,\theta \right)}{\partial r}$$

(13) $$r^{2}\frac{\partial^{2}v^{i}\left(r,\theta \right)}{\partial r^{2}}+r\frac{\partial v^{i}\left(r,\theta \right)}{\partial r}-v^{i}\left(r,\theta \right)+\left(\frac{2-2\nu^{i}}{1-2\nu^{i}}\right)\frac{\partial^{2}v^{i}\left(r,\theta \right)}{\partial \theta^{2}}+\left(\frac{1}{1-2\nu^{i}}\right)r\frac{\partial^{2}u^{i}\left(r,\theta \right)}{\partial r\partial \theta }+\left(\frac{3-4\nu^{i}}{1-2\nu^{i}}\right)\frac{\partial u^{i}\left(r,\theta \right)}{\partial \theta }=\left(\frac{2+2\nu^{i}}{1-2\nu^{i}}\right)\alpha^{i}r\frac{\partial T^{i}\left(r,\theta \right)}{\partial \theta }$$

To solve Eq. (12) and Eq. (13), the displacement components can be expressed as follows:

(14) $$\begin{array}{l} u^{i}\left(r,\theta \right)=\sum_{n=-\infty }^{\infty }u_{n}^{i}\left(r\right)e^{in\theta },v^{i}\left(r,\theta \right)=\sum_{n=-\infty }^{\infty }v_{n}^{i}\left(r\right)e^{in\theta } \end{array}$$

The co-efficient of Eq. (14) can be found in the same way as the previous technique (section 2).

Substituting Eq. (14) and Eq. (7) into Eq. (12) and Eq. (13), respectively, provides

(15) $$r^{2}\frac{d^{2}u_{n}^{i}}{dr^{2}}+r\frac{du_{n}^{i}}{dr}-\left(1+\frac{\left(1-2\nu^{i}\right)n^{2}}{2-2\nu^{i}}\right)u_{n}^{i}+\left(\frac{in}{2-2\nu^{i}}\right)r\frac{dv_{n}^{i}}{dr}+in\left(\frac{4\left(\nu^{i}-3\right)}{2-2\nu^{i}}\right)v_{n}^{i}=\left(\frac{1+\nu^{i}}{1-\nu^{i}}\right)\alpha^{i}\left(\xi_{n1}A_{n1}^{i}r^{\xi_{n1}+1}+\xi_{n2}A_{n2}^{i}r^{\xi_{n2}+1}\right)$$

(16) $$r^{2}\frac{d^{2}v_{n}^{i}}{dr^{2}}+r\frac{dv_{n}^{i}}{dr}-\left(1+\frac{\left(2-2\nu^{i}\right)n^{2}}{1-2\nu^{i}}\right)v_{n}^{i}+\left(\frac{in}{1-2\nu^{i}}\right)r\frac{du_{n}^{i}}{dr}+in\left(\frac{3-4\nu^{i}}{2-2\nu^{i}}\right)u_{n}^{i}=in\left(\frac{2+2\nu^{i}}{1-2\nu^{i}}\right)\alpha^{i}\left(A_{n1}^{i}r^{\xi_{n1}+1}+A_{n2}^{i}r^{\xi_{n2}+1}\right)$$

Eq. (15) and Eq. (16) are systems of the Cauchy-Euler equation that have general and particular solutions. The general solutions are assumed as:

(17) $$\begin{array}{l} u_{n}^{i,g}=\chi_{1}^{i}r^{\gamma^{i}}\\ v_{n}^{i,g}=\chi_{2}^{i}r^{\gamma^{i}} \end{array}$$

Eq. (17) is substituted for Eq. (15) and Eq. (16), yielding

$$\left[\gamma^{i}\left(\gamma^{i}-1\right)+\gamma^{i}-\left(1+\frac{\left(1-2\nu^{i}\right)n^{2}}{2-2\nu^{i}}\right)\right]\chi_{1}^{i}+in\;\left[\left(\frac{\gamma^{i}}{2-2\nu^{i}}\right)+\left(\frac{4\left(\nu^{i}-3\right)}{2-2\nu^{i}}\right)\right]\chi_{2}^{i}=0$$

(18) $$\left[\gamma^{i}\left(\gamma^{i}-1\right)+\gamma^{i}-\left(1+\frac{\left(2-2\nu^{i}\right)n^{2}}{1-2\nu^{i}}\right)\right]\chi_{2}^{i}+in\;\left[\left(\frac{\gamma^{i}}{1-2\nu^{i}}\right)+\left(\frac{3-4\nu^{i}}{2-2\nu^{i}}\right)\right]\chi_{1}^{i}=0$$

A nontrivial solution of Eq. (18) is obtained as:

(19) $$\left[\gamma^{i}\left(\gamma^{i}-1\right)+\gamma^{i}-\left(1+\frac{\left(1-2\nu^{i}\right)n^{2}}{2-2\nu^{i}}\right)\right]\left[\gamma^{i}\left(\gamma^{i}-1\right)+\gamma^{i}-\left(1+\frac{\left(2-2\nu^{i}\right)n^{2}}{1-2\nu^{i}}\right)\right]+n^{2}\;\left[\left(\frac{\gamma^{i}}{2-2\nu^{i}}\right)+\left(\frac{4\left(\nu^{i}-3\right)}{2-2\nu^{i}}\right)\right]\left[\left(\frac{\gamma^{i}}{1-2\nu^{i}}\right)+\left(\frac{3-4\nu^{i}}{2-2\nu^{i}}\right)\right]=0$$

Eq. (19) is a quartic equation with four roots $\gamma_{n1}^{i}\;\text{to}\;\gamma_{n4}^{i}$ that can be obtained using Ferrari's method [43].

Thus, the general solutions are

(20) $$\begin{array}{l} u_{n}^{i,g}=\sum_{j=1}^{4}\left(\chi_{1\;nj}^{i}r^{\gamma_{nj}^{i}}\right)\\ v_{n}^{i,g}=\sum_{j=1}^{4}\left(M_{nj}\chi_{1\;nj}^{i}r^{\gamma_{nj}^{i}}\right) \end{array}$$

$$\text{Where};\;M_{nj}^{i}=\frac{\left[\gamma_{nj}^{i}\left(\gamma_{nj}^{i}-1\right)+\gamma_{nj}^{i}-\left(1+\frac{\left(1-2\nu^{i}\right)n^{2}}{2-2\nu^{i}}\right)\right]}{in\;\left[\left(\frac{\gamma_{nj}^{i}}{2-2\nu^{i}}\right)+\left(\frac{4\left(\nu^{i}-3\right)}{2-2\nu^{i}}\right)\right]}$$

Particular solutions can be written as:

(21) $$\begin{array}{l} u_{n}^{i,p}=\Gamma_{n1}^{i}r^{\xi_{n1}^{i}+1}+\Gamma_{n2}^{i}\;r^{\xi_{n2}^{i}+1}\\ v_{n}^{i,p}=\Gamma_{n3}^{i}r^{\xi_{n1}^{i}+1}+\Gamma_{n4}^{i}\;r^{\xi_{n2}^{i}+1} \end{array}$$

Substitute Eq. (21) into Eqs. (15), (16), provides

(22) $$\begin{array}{l} \Psi_{1}^{i}\Gamma_{n1}^{i}r^{\xi_{n1}^{i}-1}+\Psi_{2}^{i}\Gamma_{n2}^{i}r^{\xi_{n2}^{i}-1}+\Psi_{3}^{i}\Gamma_{n3}^{i}r^{\xi_{n1}^{i}-1}+\Psi_{4}^{i}\Gamma_{n4}^{i}r^{\xi_{n2}^{i}-1}=\Psi_{5}^{i}r^{\xi_{n1}^{i}-1}+\Psi_{6}^{i}r^{\xi_{n2}^{i}-1}\\ \Psi_{7}^{i}\Gamma_{n3}^{i}r^{\xi_{n1}^{i}-1}+\Psi_{8}^{i}\Gamma_{n4}^{i}r^{\xi_{n2}^{i}-1}+\Psi_{9}^{i}\Gamma_{n1}^{i}r^{\xi_{n1}^{i}-1}+\Psi_{10}^{i}\Gamma_{n2}^{i}r^{\xi_{n2}^{i}-1}=\Psi_{11}^{i}r^{\xi_{n1}^{i}-1}+\Psi_{12}^{i}r^{\xi_{n2}^{i}-1} \end{array}$$

Where $\Psi_{j\left(j=1..12\right)}^{i}$ is given [36].

From Eq. (22) the unknown coefficient $\Gamma_{i=1..4}^{i}$ can be written as below:

$$\left\{\begin{array}{c} \Gamma_{n1}^{i}\\ \Gamma_{n2}^{i}\\ \Gamma_{n3}^{i}\\ \Gamma_{n4}^{i} \end{array}\right\}={\left[\begin{array}{cccc} \Psi_{1}^{i} & 0 & \Psi_{2}^{i} & 0\\ \Psi_{9}^{i} & 0 & \Psi_{7}^{i} & 0\\ 0 & \Psi_{2}^{i} & 0 & \Psi_{4}^{i}\\ 0 & \Psi_{10}^{i} & 0 & \Psi_{8}^{i} \end{array}\right]}^{-1}\left\{\begin{array}{c} \Psi_{5}^{i}\\ \Psi_{11}^{i}\\ \Psi_{6}^{i}\\ \Psi_{12}^{i} \end{array}\right\}$$

From Eq. (21) and Eq. (20), the sum of particular and general solutions can be written as:

(23) $$\begin{array}{l} u_{n}^{i}=\sum_{j=1}^{4}\left({\chi_{1}}_{nj}^{i}r^{\gamma_{nj}^{i}}\right)+\Gamma_{n1}^{i}r^{\xi_{n1}^{i}+1}+\Gamma_{n2}^{ii}\;r^{\xi_{n2}^{i}+1}\\ v_{n}^{i}=\sum_{j=1}^{4}\left(M_{nj}^{i}{\chi_{1}}_{nj}^{i}r^{\gamma_{nj}^{i}}\right)+\Gamma_{n3}^{i}r^{\xi_{n1}^{i}+1}+\Gamma_{n4}^{ii}\;r^{\xi_{n2}^{i}+1} \end{array}$$

For n=0, $M_{nj}$ become undefined. This is due to Eq. (15) and Eq. (16) become completely decoupled, which can be written as

(24) $$\begin{array}{l} r^{2}\frac{d^{2}u_{n}^{i}}{dr^{2}}+r\frac{du_{n}^{i}}{dr}-u_{n}^{i}=\left(\frac{1+\nu^{i}}{1-\nu^{i}}\right)\alpha^{i}\left(\xi_{n1}^{i}A_{n1}^{i}r^{\xi_{n1}^{i}+1}+\xi_{n2}^{i}A_{n2}^{i}r^{\xi_{n2}^{i}+1}\right)\\ r^{2}\frac{d^{2}v_{n}^{i}}{dr^{2}}+r\frac{dv_{n}^{i}}{dr}-v_{n}^{i}=0 \end{array}$$

The solutions of Eq. (24 a, b) are

$$\begin{array}{l} u_{0}^{i}=\sum_{j=1}^{2}\left({\chi_{1}}_{oj}^{i}r^{\gamma_{0j}^{i}}+\Gamma_{0j}^{i}r^{\xi_{0j}^{i}+1}\right)\\ v_{0}^{i}=\sum_{j=3}^{4}\left({\chi_{1}}_{oj}^{i}r^{\gamma_{0j}^{i}}\right) \end{array}$$

(25) $$\begin{array}{l} \text{Where},\xi_{0j}^{i}=\pm 1\\ \Gamma_{0j}^{i}=\frac{\left(1+\nu^{i}\right)\xi_{0j}^{i}\alpha^{i}A_{0j}^{i}}{\left(1-\nu^{i}\right)\xi_{0j}^{i}\;\left(\xi_{0j}^{i}+1\right){\left(\xi_{0j}^{i}+1\right)}^{2}}\;j=1,2 \end{array}$$

Substituting Eq. (23) and Eq. (25) into Eq. (14), yields;

(26) $$u^{i}\left(r,\theta \right)=\sum_{j=1}^{2}\left({\chi_{1}}_{oj}^{i}r^{\gamma_{0j}^{i}}+\Gamma_{0j}^{i}r^{\xi_{0j}^{i}+1}\right)+\sum_{n=-\infty ,n\neq 0}^{\infty }\;\left[\sum_{j=1}^{4}\left({\chi_{1}}_{nj}^{i}r^{\gamma_{nj}^{i}}\right)+\Gamma_{n1}^{i}r^{\xi_{n1}^{i}+1}+\Gamma_{n2}^{ii}\;r^{\xi_{n2}^{i}+1}\right]e^{in\theta }v^{i}\left(r,\theta \right)=\sum_{j=3}^{4}\left({\chi_{1}}_{oj}^{i}r^{\gamma_{0j}^{i}}\right)+\sum_{n=-\infty ,n\neq 0}^{\infty }\left[\sum_{j=1}^{4}\left(M_{nj}^{i}{\chi_{1}}_{nj}^{i}r^{\gamma_{nj}^{i}}\right)+\Gamma_{n3}^{i}r^{\xi_{n1}^{i}+1}+\Gamma_{n4}^{ii}\;r^{\xi_{n2}^{i}+1}\right]e^{in\theta }$$

The following stress distribution can be obtained from Eq. (10) by substituting Eq. (26) into Eq. (9). ${\sigma_{rr}}^{i}=\frac{E_{i}e^{in\theta }}{\left(1-2\nu^{i}\right)\left(\;1+\nu^{i}\right)}(\begin{array}{l} \sum_{j=1}^{2}\left(\left(1-\nu^{i}\right)\gamma_{0j}^{i}+\nu^{i}\right){\chi_{1}}_{0j}^{i}r^{\gamma_{0j}^{i}-1}+\left(\nu^{i}\xi_{oj}^{i}+1-\left(1-\nu^{i}\right)\alpha_{i}\right)\Gamma_{oj}^{i}r^{\xi_{0j}^{i}}\\ +\sum_{n=-\infty ,n\neq 0}^{\infty }[\sum_{j=1}^{4}\left(\left(1-\nu^{i}\right)\gamma_{nj}^{i}+\nu^{i}\left(inM_{nj}^{i}+1\right)\right){\chi_{1}}_{nj}^{i}r^{\gamma_{nj}^{i}-1}+(\left(1-\nu^{i}\right)\left(\xi_{n1}^{i}+1\right)\Gamma_{n2}^{i}\\ +\nu^{i}\;\left(in\;\Gamma_{n3}^{i}+\Gamma_{n1}^{i}\right)-\left(1+\nu^{i}\right)\alpha^{i}A_{n1}^{i})r^{\xi_{n1}^{i}}+(\left(1-\nu^{i}\right)\left(\xi_{n2}^{i}+1\right)\Gamma_{n2}^{i}\\ +\nu^{i}\left(in\Gamma_{n4}^{i}+\Gamma_{n2}^{i}\right)-\left(1+\nu^{i}\right)\alpha^{i}A_{n2}^{i})r^{\xi_{n2}^{i}}]) \end{array})$.

$${\sigma_{\theta \theta }}^{i}=\frac{E_{i}e^{in\theta }}{\left(1-2\nu^{i}\right)\left(\;1+\nu^{i}\right)}\left(\begin{array}{l} \sum_{j=1}^{2}\left(\left(1-\nu^{i}\right)\gamma_{oj}^{i}+\nu^{i}\right){\chi_{1}}_{0j}^{i}r^{\gamma_{0j}^{i}-1}+(\left(1-\nu^{i}\right)\left(\xi_{oj}^{i}+1-\left(1+\nu^{i}\right)\alpha_{i}\right)\Gamma_{oj}^{i}r^{\xi_{0j}^{i}}\\ +\sum_{n=-\infty ,n\neq 0}^{\infty }[\sum_{j=1}^{4}\left(\nu^{i}\gamma_{nj}^{i}+\left(1-\nu^{i}\right)\left(inM_{nj}^{i}+1\right)\right){\chi_{1}}_{nj}^{i}r^{\gamma_{nj}^{i}}+(\nu^{i}\left(\xi_{n1}^{i}+1\right)\Gamma_{n1}^{i}\\ +\left(1-\nu^{i}\right)\;\left(in\;\Gamma_{n3}^{i}+\Gamma_{n1}^{i}\right)-\left(1+\nu^{i}\right)\alpha_{i}A_{n1}^{i})r^{\xi_{n1}^{i}}+(\nu^{i}\left(\xi_{n2}^{i}+1\right)\Gamma_{n2}^{i}\\ +\left(1-\nu^{i}\right)\left(in\Gamma_{n3}^{i}+\Gamma_{n2}^{i}\right)-\left(1+\nu^{i}\right)\alpha_{i}A_{n2}^{i})r^{\xi_{n2}^{i}}]) \end{array}\right)$$

(27) $${\sigma_{r\theta }}^{i}=\frac{E_{i}}{\left(\;1+\nu^{i}\right)}\left(\begin{array}{l} \left(\gamma_{04}^{i}-1\right){\chi_{1}}_{04}^{i}r^{\gamma_{o4}^{i}-1}\\ +\sum_{n=-\infty ,n\neq 0}^{\infty }\left[\sum_{j=1}^{4}\begin{array}{l} (in+\left(\gamma_{nj}^{i}-1\right)M_{nj}^{i}){\chi_{1}}_{nj}^{i}r^{\gamma_{nj}^{i}-1}+\left(in\Gamma_{n1}^{i}+\xi_{n1}^{i}\Gamma_{n3}^{i}\right)r^{\xi_{n1}^{i}}\\ +\left(in\Gamma_{n2}^{i}+\xi_{n2}^{i}\Gamma_{n4}^{i}\right)r^{\xi_{n1}^{i}} \end{array}\right] \end{array}\right)$$

$\chi_{n1}^{1},\chi_{n2}^{1},\chi_{n3}^{1},\chi_{n4}^{1},\chi_{n1}^{2},\chi_{n2}^{2},\chi_{n3}^{2},\chi_{n4}^{2}$ are the total eight unknowns that need to be found. As the cylinder is considered perfectly jointed the continuity requirement of displacement and stress are

(28) $$\begin{array}{l} {u^{i}\left(b,\theta \right)|}_{i=1}={u^{i}\left(b,\theta \right)|}_{i=2}\\ {v^{i}\left(b,\theta \right)|}_{i=1}={v^{i}\left(b,\theta \right)|}_{i=2}\\ {{\sigma_{rr}}^{i}\left(b,\theta \right)|}_{i=1}={{\sigma_{rr}}^{i}\left(b,\theta \right)|}_{i=2}\\ {{\sigma_{r\theta }}^{i}\left(b,\theta \right)|}_{i=1}={{\sigma_{r\theta }}^{i}\left(b,\theta \right)|}_{i=2} \end{array}$$

All possible mechanical boundary conditions can be stated as

(29) $$\begin{array}{l} u^{i}\left(a,\theta \right)=\Phi_{1}\left(\theta \right)\\ v^{i}\left(a,\theta \right)=\Phi_{2}\left(\theta \right)\\ u^{i}\left(c,\theta \right)=\Phi_{3}\left(\theta \right)\\ v^{i}\left(c,\theta \right)=\Phi_{4}\left(\theta \right)\\ \sigma_{rr}^{i}\left(a,\theta \right)=\Phi_{5}\left(\theta \right)\\ \sigma_{rr}^{i}\left(c,\theta \right)=\Phi_{6}\left(\theta \right)\\ \sigma_{r\theta }^{i}\left(a,\theta \right)=\Phi_{7}\left(\theta \right)\\ \sigma_{r\theta }^{i}\left(c,\theta \right)=\Phi_{8}\left(\theta \right) \end{array}$$

As a result, the integration constant can be solved using Eq. (28) and any Eq. (29) (choosing any four combinations).

To determine the integration constant, B.C. must expand in a complex Fourier series as

$$\begin{array}{l} \Phi_{i}\left(\theta \right)=\sum_{n=-\infty }^{\infty }\Omega_{i}\left(n\right)e^{in\theta }\;i=1..4\\ \text{Where},\Omega_{i}\left(n\right)\;\text{is}\;\text{co}-\text{efficient}\;\text{of}\;\text{complex}\;\text{Fourier}\;\text{series} \end{array}$$

## 4 Finite difference method (FDM) solution

In order to verify the analytical solution, a numerical finite difference method (FDM) is obtained. Here, the partial differential equation of energy equation and equations of motion are solved numerically using second-order accuracy $o\left(h^{2}\right)\;\text{and}\;o\left(k^{2}\right)$.

### 4.1 Solution of the energy equation

Eq. (1) can be discretized utilizing Table 1.

(30) $$\alpha_{m}T_{m+1,n}^{i}+\beta_{m}T_{m,n}^{i}+\delta_{m}T_{m-1,n}^{i}+\gamma_{m}T_{m-1,n}^{i}+\gamma_{m}T_{m,n-1}^{i}=0$$

$$\begin{array}{l} \text{Where,}\\ \left\{ \begin{array}{l} \alpha_{m}=\left[\frac{1}{h^{2}}+\frac{1}{2r_{m}h}\right]\\ \beta_{m}=-\left[\frac{1}{h^{2}}+\frac{1}{r_{m}^{2}k^{2}}\right]\\ \delta_{m}=\left[\frac{1}{h^{2}}-\frac{1}{2r_{m}h}\right]\\ \gamma_{m}=\frac{1}{r_{m}^{2}k^{2}} \end{array}\right. \end{array}$$

**Table 1:** Finite difference expressions for first and second derivatives for the energy equation.

Derivative	Formula	Nodes
	
${\frac{\partial T^{i}\left(r,\theta \right)}{\partial r}|}_{C.D}$	$\frac{T_{m+1,n}^{i}-T_{m-1,n}^{i}}{2h}$	Intermediate nodes
${\frac{\partial T^{i}\left(r,\theta \right)}{\partial \theta }|}_{C.D}$	$\frac{T_{m,n+1}^{i}-T_{m,n-1}^{i}}{2k}$	Intermediate nodes
${\frac{\partial^{2}T^{i}\left(r,\theta \right)}{\partial r^{2}}|}_{C.D}$	$\frac{T_{m+1,n}^{i}-T_{m,n}^{i}+T_{m-1,n}^{i}}{h^{2}}$	Intermediate nodes
${\frac{\partial^{2}T^{i}\left(r,\theta \right)}{\partial \theta^{2}}|}_{C.D}$	$\frac{T_{m,n+1}^{i}-T_{m,n}^{i}+T_{m,n-1}^{i}}{k^{2}}$	Intermediate nodes
${\frac{\partial T^{i}\left(r,\theta \right)}{\partial r}|}_{F.D}$	$\frac{-3T_{m,n}^{i}+4T_{m+1,n}^{i}-T_{m+2,n}^{i}}{2h}$	First nodes (Inner surface)
${\frac{\partial T^{i}\left(r,\theta \right)}{\partial r}|}_{B.D}$	$\frac{T_{m-2,n}^{i}-4T_{m-1,n}^{i}+T_{m,n}^{i}}{2h}$	Last nodes (Outer surface)

Again, using Table 1, the spatially discretized equation derived from B.C.s Eq. (2 a, b) and Eq. (8 a, b) can be expressed as follows:

$$\left(C_{11}-\frac{3}{2}\frac{C_{12}}{h}\right)T_{m,n}^{i}+\frac{2C_{12}}{h}T_{m+1,n}^{i}-\frac{1}{2}\frac{C_{12}}{h}T_{m+2,n}^{i}=f_{1}\left(\theta_{k}\right)\;\text{When},m=1$$

$$\frac{1}{2}\frac{C_{22}}{h}T_{m-2,n}^{i}-\frac{2C_{22}}{h}T_{m-1,n}^{i}+\left(C_{21}+\frac{3}{2}\frac{C_{22}}{h}\right)T_{m,n}^{i}=f_{2}\left(\theta_{k}\right)\;\text{When},m=N$$

$${T_{l,n}^{i}|}_{i=1}={T_{l,n}^{i}|}_{i=2}$$

(31) $$T_{l-2,n}^{i=1}-4T_{l-1,n}^{i=1}+3\left(1+\frac{k^{i=2}}{k^{i=1}}\right)T_{l,n}^{i=1}-4\left(\frac{k^{i=2}}{k^{i=1}}\right)T_{l+1,n}^{i=2}+\frac{k^{i=2}}{k^{i=1}}T_{l+2,n}^{2}=0$$

where *l* is the location at the contact surface of two different isotropic materials.

Eqs. (30) and (31) produce a system of linear equations with unknown temperatures $T_{m,n}^{i}$ that may be used to generate TDM, which can be solved using the well-known Thomas algorithm.

### 4.2 Solution of the equation of motion

From Eqs. (12), (13), it constitutes a system of coupled linear partial differential equations that can be discretized utilizing Table 2, as shown below.

$$\tau_{11}u_{m+1,n}^{i}+\tau_{12}u_{m,n}^{i}+\tau_{13}u_{m-1,n}^{i}+\tau_{14}\left(u_{m,n+1}^{i}+u_{m,n-1}^{i}\right)+\tau_{15}\left(v_{m-1,n-1}^{i}-v_{m-1,n+1}^{i}-v_{m+1,n-1}^{i}+v_{m+1,n+1}^{i}\right)+\tau_{16}\left(v_{m,n+1}^{i}-v_{m,n-1}^{i}\right)=\tau_{17}\left(T_{m+1,n}^{i}-T_{m-1,n}^{i}\right)$$

(32) $$\tau_{21}v_{m+1,n}^{i}-\tau_{22}v_{m,n}^{i}+\tau_{23}u_{m-1,n}^{i}+\tau_{24}\left(v_{m,n+1}^{i}+v_{m,n-1}^{i}\right)+\tau_{25}\left(u_{m-1,n-1}^{i}-u_{m-1,n+1}^{i}-u_{m+1,n-1}^{i}+u_{m+1,n+1}^{i}\right)+\tau_{26}\left(u_{m,n+1}^{i}-u_{m,n-1}^{i}\right)=\tau_{27}\left(T_{m,n+1}^{i}-T_{m,n-1}^{i}\right)$$

**Table 2:** Finite differences equation of for equation of motion.

Derivative	Formula	Nodes
	
${\frac{\partial u^{i}\left(r,\theta \right)}{\partial r}|}_{C.D}$	$\frac{u_{m+1,n}^{i}-u_{m-1,n}^{i}}{2h}$	Intermediate nodes
${\frac{\partial v^{i}\left(r,\theta \right)}{\partial r}|}_{C.D}$	$\frac{v_{m+1,n}^{i}-v_{m-1,n}^{i}}{2h}$	Intermediate nodes
${\frac{\partial u^{i}\left(r,\theta \right)}{\partial \theta }|}_{C.D}$	$\frac{u_{m,n+1}^{i}-u_{m,n-1}^{i}}{2k}$	Intermediate nodes
${\frac{\partial v^{i}\left(r,\theta \right)}{\partial \theta }|}_{C.D}$	$\frac{v_{m,n+1}^{i}-v_{m,n-1}^{i}}{2k}$	Intermediate nodes
${\frac{\partial^{2}u^{i}\left(r,\theta \right)}{\partial r^{2}}|}_{C.D}$	$\frac{u_{m+1,n}^{i}-u_{m,n}^{i}+u_{m-1,n}^{i}}{h^{2}}$	Intermediate nodes
${\frac{\partial^{2}v^{i}\left(r,\theta \right)}{\partial r^{2}}|}_{C.D}$	$\frac{v_{m+1,n}^{i}-v_{m,n}^{i}+v_{m-1,n}^{i}}{h^{2}}$	Intermediate nodes
${\frac{\partial^{2}u^{i}\left(r,\theta \right)}{\partial \theta^{2}}|}_{C.D}$	$\frac{u_{m,n+1}^{i}-u_{m,n}^{i}+u_{m,n-1}^{i}}{k^{2}}$	Intermediate nodes
${\frac{\partial^{2}v^{i}\left(r,\theta \right)}{\partial \theta^{2}}|}_{C.D}$	$\frac{v_{m,n+1}^{i}-v_{m,n}^{i}+v_{m,n-1}^{i}}{k^{2}}$	Intermediate nodes
${\frac{\partial^{2}u^{i}\left(r,\theta \right)}{\partial \theta \partial r}|}_{C.D}$	$\frac{u_{m-1,n-1}^{i}-u_{m-1,n+1}^{i}-u_{m+1,n-1}^{i}+u_{m+1,n+1}^{i}}{4hk}$	Intermediate nodes
${\frac{\partial^{2}v^{i}\left(r,\theta \right)}{\partial \theta \partial r}|}_{C.D}$	$\frac{v_{m-1,n-1}^{i}-v_{m-1,n+1}^{i}-v_{m+1,n-1}^{i}+v_{m+1,n+1}^{i}}{4hk}$	Intermediate nodes
${\frac{\partial T^{i}\left(r,\theta \right)}{\partial r}|}_{F.D}$	$\frac{-3T_{m,n}^{i}+4T_{m+1,n}^{i}-T_{m+2,n}^{i}}{2h}$	First nodes (Inner surface)
${\frac{\partial T^{i}\left(r,\theta \right)}{\partial r}|}_{B.D}$	$\frac{T_{m-2,n}^{i}-4T_{m-1,n}^{i}+T_{m,n}^{i}}{2h}$	Last nodes (Outer surface)
${\frac{\partial u^{i}\left(r,\theta \right)}{\partial r}|}_{F.D}$	$\frac{-3u_{m,n}^{i}+4u_{m+1,n}^{i}-u_{m+2,n}^{i}}{2h}$	First nodes (Inner surface)
${\frac{\partial u^{i}\left(r,\theta \right)}{\partial r}|}_{B.D}$	$\frac{u_{m-2,n}^{i}-4u_{m-1,n}^{i}+u_{m,n}^{i}}{2h}$	Last nodes (Outer surface)

where the parameters are given in Appendix A.

There are two types of derivative boundary conditions (secondary variables): one is normal to the surface, and the other is tangential. The asymmetric normal B.C.s (pressure loading) shown here is an example of the first type.

From Eq. (10 a) and Eq. (28), the discretized form of the inner surface and outer surface pressure loading with interfacial conditions can be shown in the set of algebraic equations.

$$\begin{array}{l} \Omega_{11}u_{m,n}^{i}-\Omega_{12}u_{m+1,n}^{i}+\Omega_{13}u_{m+2,n}^{i}+\Omega_{14}\left(v_{m,n-1}^{i}-v_{m,n+1}^{i}\right)+\Omega_{15}T_{m,n}^{i}+P_{m,n}^{i}=0∵i,m=1\\ \Omega_{21}u_{m-2,n}^{i}+\Omega_{22}u_{m,n}^{i}-\Omega_{23}u_{m+2,n}^{i}+\Omega_{14}\left(v_{m,n-1}^{i}-v_{m,n+1}^{i}\right)+\Omega_{15}T_{m,n}^{i}+P_{m,n}^{i}=0∵i=2,m=N \end{array}$$

$$\begin{array}{l} {u_{l,n}^{i}|}_{i=1}={u_{l,n}^{i}|}_{i=2}\\ {v_{l,n}^{i}|}_{i=1}={v_{l,n}^{i}|}_{i=2} \end{array}$$

(33) $$\begin{array}{l} \Theta_{11}u_{l-2,n}^{i=1}-\Theta_{12}u_{l-1,n}^{i=1}+\Theta_{13}u_{l,n}^{i=1}+\Theta_{14}\left(v_{l,n+1}^{i=1}-v_{l,n-1}^{i=1}\right)+\Theta_{15}=-\Theta_{21}u_{l+2,n}^{i=2}+\Theta_{22}u_{l+1,n}^{i=2}-\Theta_{23}u_{l,n}^{i=2}+\Theta_{24}\left(v_{l,n+1}^{i=2}-v_{l,n-1}^{i=2}\right)+\Theta_{25}\\ \iota_{11}\left(u_{l,n+1}^{i=1}-u_{l,n-1}^{i=1}\right)+\iota_{12}v_{l-2,n}^{i=1}-\iota_{13}v_{l-1,n}^{i=1}+\iota_{14}v_{l,n}^{i=1}=\iota_{21}\left(u_{l,n+1}^{i=2}-u_{l,n-1}^{i=2}\right)-\iota_{22}v_{l+2,n}^{i=2}+\iota_{23}v_{l+1,n}^{i=2}-\iota_{24}v_{l,n}^{i=2} \end{array}$$

Eqs. (32) and (33) formed a set of coupled algebraic equations that can be solved using inner and outer iterations in the Picard method.

After the solution for u,v then, stresses can be computed from Eq. (10 a, b, c).

However, the computational resources, such as time and memory, needed to solve the steady-state problem through the proposed method for a cylindrical body are influenced by several factors. These factors encompass the dimensions of the problem, commonly referred to as the mesh size, the choice of numerical solver, and the unique attributes of the material and boundary conditions involved. The suggested technique effectively simplifies the mathematical complexity and optimizes the grid size. Consequently, the memory and time requirements are significantly reduced compared to other techniques such as FEM, FVM, DQM. In a nutshell the suggested technique demonstrates good performance in several aspects, including computing time, memory consumption, scalability, accuracy and precision, robustness, implementation and user-friendliness, parallelization, and resource requirements.

## 5 Results and discussions

### 5.1 Verification and comparative study

Since this problem does not yet have a solution, validation is acquired through exact and numerical comparison. However, the suggested analytical approach and numerical method (FDM) are in good agreement with each other, with a maximum error of 1.53 % indicated in Table-5. This agreement confirms the validity of the presented method.

**Fig. 2:**
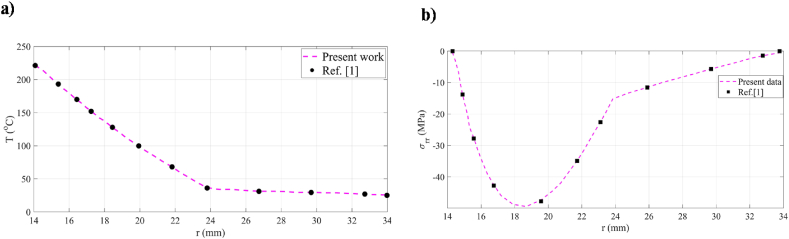
Comparison between current work and Ref. [1].

In order to validity of proposed methods, it is decided to compare the current results with reference [1] under symmetric conditions because it has been previously stated that no research has been done on compound metallic cylinders under asymmetric loads. It has been shown (Fig. 2) that the currently proposed methods have strong agreement with those obtained from Ref. [1].

### 5.2 Current results

The analysis of compound cylinder strength and compliance, influenced by the usage conditions, is visualized by observing stress and displacement patterns across the varying layer's thickness (r) and tangential angle (θ)**.** Table 3 displays the mechanical characteristics of the given setup (Fig. 1) that affect the output field. Here, two material configurations (steel-aluminum) are used to demonstrate the above method. In addition, the dimensions of the used system are: c=3a,b=2a. To facilitate the illustration, the discussion can be divided into three cases. Each case emphasizes various types of possible B.C.s, showing the variability of the problems.

CASE 1 Only thermal loading

**Fig. 3:**
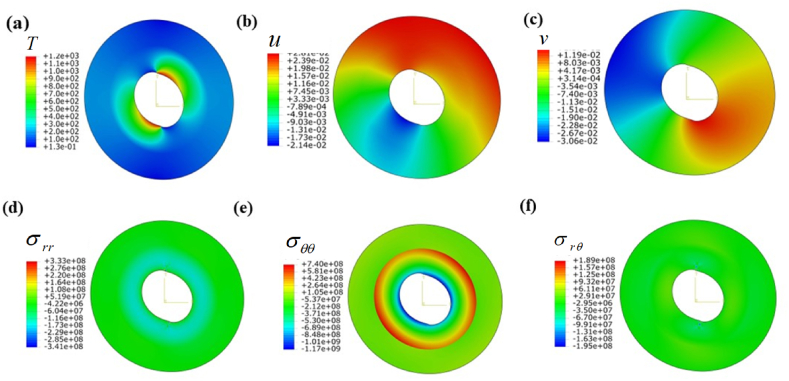
Contour plot under Dirichlet B.C.s when the cylinder is subjected to only thermal load: (a) Temperature field T(r,θ), (b) radial displacement field u(r,θ), (c) tangential displacement field v(r,θ), (d) radial stress field, $\sigma_{rr}\left(r,\theta \right)$, (e) tangential stress field, $\sigma_{\theta \theta }\left(r,\theta \right)$, and (f) shear stress field in (r−θ) plane.

In this numerical approach, the compound cylinder is subjected to three types of thermal boundary conditions shown in Table 4. Fig. 3 a shows the temperature field under Dirichlet-type boundary conditions. The above figure shows a good agreement with the given boundary conditions (Table 4). However, the maximum temperature zone is $\theta =0^{o}\;\text{to}\;90^{o}$ in the first cylinder, and then a sharp decrease in temperature is extended to the second cylinder. Furthermore, temperature distribution becomes symmetrical $\theta =135^{o}$. Temperature variations result in the emergence of thermal stresses, leading to the occurrence of thermal strain and subsequent displacements. Given that this is a two-dimensional problem, there are displacement fields depicted for radial (u) and tangential (v) directions in Fig. 3b and Fig. 3c, respectively. The upper section $\left(\theta =-35^{o}\;\text{to}\;135^{o}\right)$ of the cylinder exhibits the maximum radial displacement, while the lower portion $\left(\theta =180^{o}\;\text{to}\;270^{o}\right)$ experiences a negative displacement field. Moreover, In Fig. 3 c, the tangential displacement (v) reaches its maximum value at $\theta =30^{o}\;\text{to}\;270^{o}$. Similarly, the opposing section encounters a displacement in the negative direction. Because of the non-uniformity of the boundary conditions, the entire domain exhibits irregular contours of displacement. The radial stress $\left(\sigma_{rr}\right)$ displays an axisymmetric characteristic, gradually diminishing in the thickness direction as depicted in Fig. 3 d. On the contrary, the hoop stress $\left(\sigma_{\theta \theta }\right)$ demonstrates a rapid decrease at the interfacial surface, as illustrated in Fig. 3 e. This is due to the sudden change of material properties at the joint. The thermal stress results in a consistent shear stress across the entire domain, as illustrated in Fig. 3 f. This is represented by a shear strain field that is considered negligible.

**Table 4:** Assumptions and boundary conditions (B.C.s).

Types of loading	Boundary conditions (B.C.s)	Inner surface	Outer surface
Only thermal load	Dirichlet B.C.	T(a,θ)=600(1+sin(θ))°C	T(c,θ)=200(cos(θ))°C
	Neumann B.C.	$q\left(a,\theta \right)=1357\left(1+\cos\left(\frac{\theta }{2}\right)\right)\;W/m^{2}k$	T(c,θ)=200(cos(θ))°C
	Robins B.C.	$\begin{array}{l} h\left(a,\theta \right)=200\left(1+\cos\left(\frac{\theta }{5}\right)\right)W/m^{2}k\\ T_{\propto_{1}}\left(\theta \right)=600\left(1+\sin\left(2\theta \right)\right){}^{\circ}C \end{array}$	$\begin{array}{l} h\left(c\right)=100\;W/m^{2}k\\ T_{\propto_{2}}\left(c\right)=200{}^{\circ}C \end{array}$
Only mechanical load	Pressure B.C	$\sigma_{rr}\left(a,\theta \right)=400\;\sin\left(\frac{\theta^{2}}{4}-\theta \right)$ MPa	u=v=0
	Shear B.C	$\sigma_{r\theta }\left(a,\theta \right)=50\;\theta^{2}\;\cos\left(\theta \right)\;MPa$	u=v=0
Thermo-mechanical load	Thermal B.C.	$\begin{array}{l} h\left(a,\theta \right)=200\left(1+\cos\left(\frac{\theta }{5}\right)\right)\;W/m^{2}k\\ T_{\propto_{1}}\left(\theta \right)=600\left(1+\sin\left(2\theta \right)\right){}^{\circ}C \end{array}$	$q\left(\theta \right)=1357\left(1+\cos\left(\frac{\theta }{2}\right)\right)$
	Mechanical B.C.	$\begin{array}{l} \sigma_{rr}\left(a,\theta \right)=400\;\sin\left(\frac{\theta^{2}}{4}-\theta \right)\;MPa\\ \sigma_{r\theta }\left(a,\theta \right)=50\;\theta^{2}\;\cos\left(\theta \right)\;MPa \end{array}$	u=v=0

**Table 5:** Comparison of analytical solution with FDM solution when $\theta =0^{o}$ under thermomechanical loading (Table 4).

$\left(\frac{r-a}{c-a}\right)$	Types	$\frac{T}{T_{i}}$		u(mm)		v(mm)		$\frac{\sigma_{rr}}{P_{i}}$		$\frac{\sigma_{\theta \theta }}{P_{i}}$		$\frac{\sigma_{r\theta }}{\tau_{o}}$	
		Val.	%Er.	Val.	%Er.	Val.	%Er.	Val.	%Er.	Val.	%Er.	Val.	%Er.
0	Anal.	1.03	0	−25.8	0	−5.43	0	−0.06	0	−20.3	0.49	1.03	0
	FDM	1.03		−25.8		−5.43		−0.06		−20.4		1.03	
0.19	Anal.	1.22	0.80	−18.6	0.53	−3.59	0.27	**−**4.97	0.40	−16.5	0.60	3.47	0.28
	FDM	1.23		−18.7		−3.60		−4.99		−16.6		3.48	
0.38	Anal.	1.36	1.44	−13.6	1.44	−2.41	0.41	−7.26	0.27	−15	1.31	2.43	0.81
	FDM	138		−13.8		−2.42		−7.28		−15.2		2.45	
0.56	Anal.	1.44	1.36	−9.22	0.10	−1.54	0.64	−8.19	0.24	−10.8	0.91	1.28	1.53
	FDM	1.46		−9.23		−1.55		−8.21		−10.9		1.30	
0.75	Anal.	1.45	1.37	−4.87	0.40	−0.72	2.77	−8.58	0.23	−10.5	0.94	0.81	1.21
	FDM	1.47		−4.89		−0.74		−8.60		−10.6		0.82	
1	Anal.	1.46	0	0.00	0	0.00	0	−8.82	0	−10.2	1.92	0.63	0
	FDM	1.46		0.00		0.00		−8.82		−10.4		0.63

**Fig. 4:**
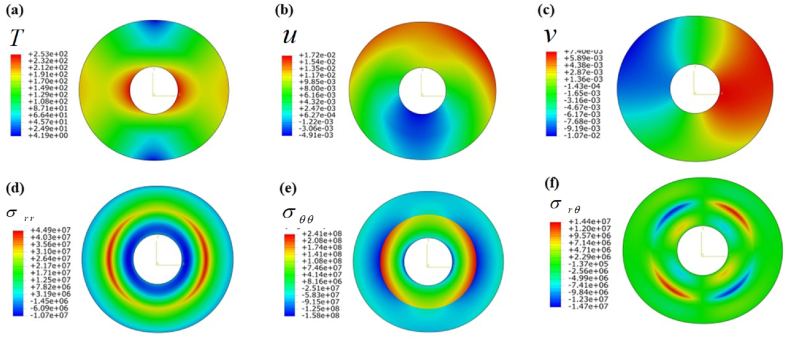
Contour plot under Neumann B.C.s when the cylinder is subjected to only thermal load: (a) Temperature field T(r,θ), (b) radial displacement field u(r,θ), (c) tangential displacement field v(r,θ), (d) radial stress field, $\sigma_{rr}\left(r,\theta \right)$ (e) tangential stress field $\sigma_{\theta \theta }\left(r,\theta \right)$, and (f) shear stress field in (r−θ) plane.

Fig. 4 Represents output fields under Neumann B.C. (Table 4). The figures imply that the temperature distribution is symmetrical about $\theta =90^{0}/0^{o}$ (Fig. 4a). The maximum temperature zone appeared $\theta =\left[-60^{o},60^{o}\right]$ and $\theta =\left[-120^{o},240^{o}\right]$ at r=[0,1.5]. However, the temperature sharply decreases at the contact points due to sudden variations in thermal conductivity. In Fig. 4 b, the radial displacement field is depicted, highlighting the peak displacement $\left(u_{\max}=17.2\;mm\right)$ occurring at the plane of $\theta \approx 170^{0}$ and indicating the smallest displacement magnitude $u_{\min}=4.91\;mm$ in the lower section of the cylinder. Nevertheless, the tangential displacement exhibits distinct patterns, as illustrated in Fig. 4 c. The maximum tangential displacement observed on the cylinder's front side is $v_{\max}=7.40\;mm$ and the minimum displacement on the cylinder's backside $v_{\min}=1.07\;mm$. In Fig. 4 d, the innermost layer is depicted with the highest compressive stress, while the point of contact experiences the maximum tensile stress. However, the mismatched properties at contact points can result in abrupt shifts in tensile stress, posing a potential risk to the structural integrity of vessel designs. As noted earlier, a similar occurrence has been observed concerning hoop stress distribution, as depicted in Fig. 4 e. Owing to periodic boundary conditions, a distinct pattern of the shear stress field has emerged. The majority of the region demonstrates consistent stress levels, with the notable exceptions of a peak stress ${\sigma_{r\theta }}_{,\max}$ at $\theta ≃\left[20^{o},80^{o}\right]\cup \left[180^{o},260^{o}\right]$ and a minimum stress ${\sigma_{r\theta }}_{,\min}$ at $\theta ≃\left[100^{o},160^{o}\right]\cup \left[280^{o},350^{o}\right]$.

**Fig. 5:**
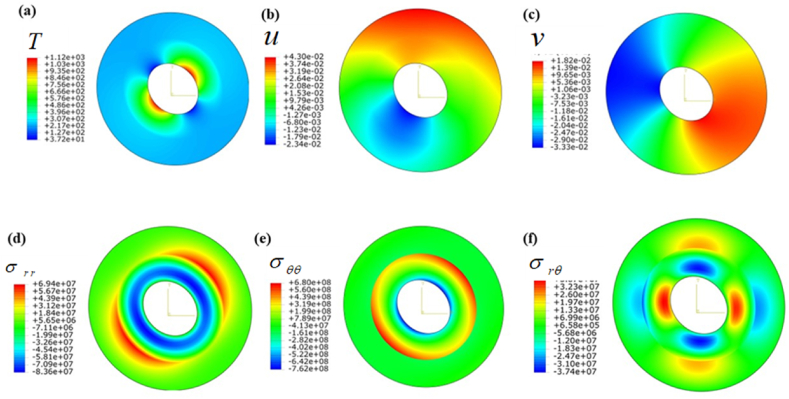
Contour plot under Robins B.C.s when the cylinder is subjected to only thermal load:(a) Temperature field T(r,θ),(b) radial displacement field u(r,θ), (c) tangential displacement field v(r,θ), (d) radial stress field, $\sigma_{rr}\left(r,\theta \right)$, (e) tangential stress field $\sigma_{\theta \theta }\left(r,\theta \right)$, and (f) shear stress field in (r−θ) plane.

Fig. 5 displays the output fields under the influence of asymmetric convection boundary conditions, as outlined in Table 4. Fig. 5 a displays temperature fields that demonstrate the occurrence of maximum temperature at $\theta ≃\left[30^{o},60^{o}\right]\cup \left[210^{o},240^{o}\right]$. Nevertheless, the periodic characteristics of the temperature field's profile conform to the requirements of periodic boundary conditions. The radial (*u*) and circumferential (*v*) displacements have been shown in Fig. 5 b and Fig. 5 c. While Fig. 5 d and 5. e depict the distribution of radial and circumferential stresses, respectively. These stress patterns give rise to periodic thermal stresses in the corresponding directions. Furthermore, it has been seen that Fig. 5 f exhibits an intricate shear stress pattern. A shear stress pocket formation, characterized by both maximum (38.4 MPa) and minimum (37.4 MPa) values, has been observed in both the inner and outer regions.

CASE 2 Only mechanical loading

• **Normal loading**

**Fig. 6:**
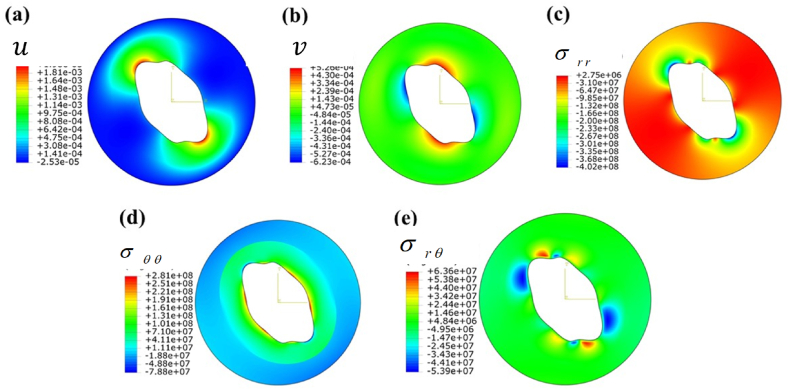
Contour plot under pressure loading when the cylinder is subjected to only mechanical load:(a) radial displacement field u(r,θ) , (b) tangential displacement field v(r,θ), (c) radial stress field, $\sigma_{rr}\left(r,\theta \right)$, (d) tangential stress field, $\sigma_{\theta \theta }\left(r,\theta \right)$, and (e) shear stress field in (r−θ) plane.

The illustration of the dependent fields under asymmetric pressure loading is presented in Fig. 6.

Fig. 6a and Fig. 6b show the large elastic deformation in the radial and tangential directions.

The radial displacement $\left(u_{\max}\right)$ reaches a peak value of 1.85 mm, while the tangential displacement registers at $v_{\max}$ = 0.52 mm at $\theta =90^{o}\cap 270^{o}$. Fig. 6 c and 6. d depict the distribution of radial and tangential stresses. The radial stress across the cylinder remains relatively consistent, except for the existence of stress discontinuities in the upper and lower regions. The hoop stress exhibits an elevated value at the inner surface, gradually decreasing through the thickness. However, the highest hoop stress is documented at 281 MPa, while the maximum radial stress is noted at 2.75 MPa.

**Table 3:** Properties of materials [1].

Material	$E^{i}\left(\text{GPa}\right)$	$\nu^{i}$	$\alpha^{i}\left(C^{-1}\right)$	$K^{i}\left(Wm^{-1}k^{-1}\right)$
Steel ASTM A564 H1150	210	0.31	$11.7\times 10^{-6}$	20
Aluminum 1050A-H9	73	0.33	$24.2\times 10^{-6}$	234

#### 5.2.1 Shear loading

**Fig. 7:**
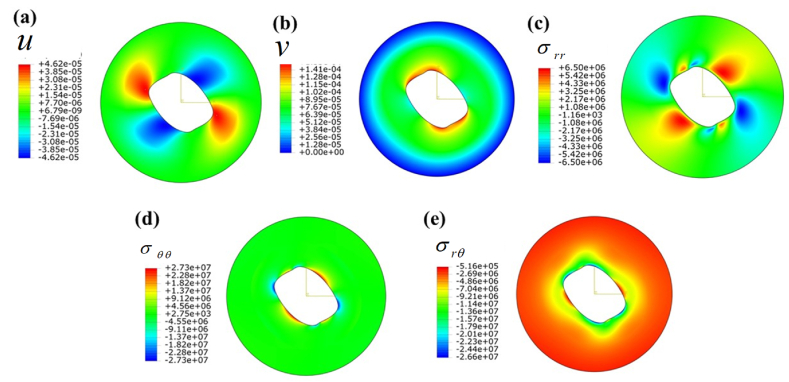
Contour plot under shear loading when the cylinder is subjected to only mechanical load:(a) radial displacement field u(r,θ), (b) tangential displacement field v(r,θ), (c) radial stress field, $\sigma_{rr}\left(r,\theta \right)$, (d) tangential stress field, $\sigma_{\theta \theta }\left(r,\theta \right)$, and (e) shear stress field in (r−θ) plane.

Fig. 7 illustrates the displacement and stress distribution observed under shear loading conditions. A pocket-shaped localized radial displacement is close to the cylinder, as depicted in Fig. 7 a. Furthermore, Fig. 7 b illustrates the distribution of tangential displacement under the shear loading conditions specified in Table 4. However, it is noted that the innermost layer has $v_{\max}=0.142mm$ at $\theta ≃\left[90^{o},45^{o}\right]\cup \left[270^{o},310^{o}\right]$. Furthermore, it is noteworthy that the tangential displacement at the outer surface is determined to be zero, thereby fulfilling the boundary condition. An analogous localized pocket of compressive and tensile radial stress has been generated, as depicted in Fig. 7 c. In Fig. 7d, a fixed tangential stress is observed to be developed. In Fig. 7 e, it is evident that the outer layer demonstrates a considerably greater shear stress compared to the inner layer.

CASE 3 Thermomechanical loading

**Fig. 8:**
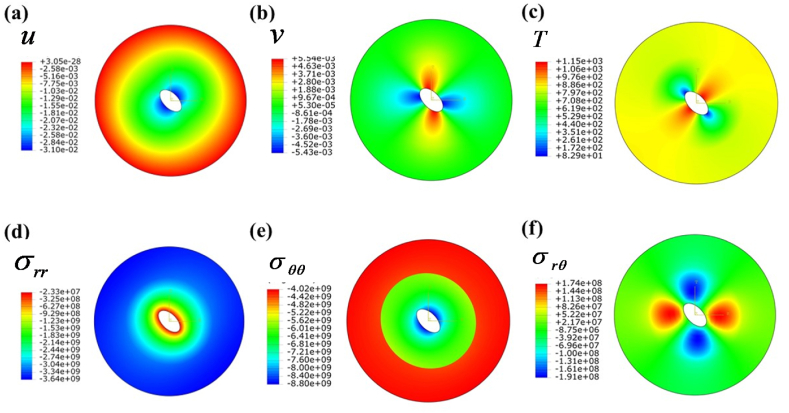
Contour plot under normal (pressure) and traction (shear) B.C.s when the cylinder is subjected to only thermo-mechanical load: (a) radial displacement field u(r,θ), (b) tangential displacement field v(r,θ), (c) Temperature field T(r,θ), (d) radial stress field, $\sigma_{rr}\left(r,\theta \right)$, (e) tangential stress field, $\sigma_{\theta \theta }\left(r,\theta \right)$, and (f) shear stress field in (r−θ) plane.

The combined effects of thermal and mechanical loading on displacement, temperature, and stress fields are illustrated in Fig. 8, with the complex operational boundary conditions detailed in Table 4. Fig. 8a depicts the radial displacement field, with the inner surface exhibiting the highest induced displacement. The outer surface exhibits zero displacement, indicating that the mechanical boundary condition has been met. In Fig. 8b, there is an observed localized tangential displacement field at the inner surface, extending from a specific radial location.

The highest temperature attained at the region $\theta ≃\left[20^{o},65^{o}\right]\cup \left[195^{o},265^{o}\right]$ and the lowest temperature observed in the vicinity of the region $\theta =145^{o}$ shown in Fig. 8 c. Fig. 8 d illustrates the radial stress distribution resulting from the specified thermomechanical boundary conditions. The observed phenomenon illustrates the initiation of radial stress at the inner surface, which then diminishes as the radial position extends toward the outer region. Fig. 8 e shows hoop stress distribution in r−θ plane. The stress at the contact region experienced a sudden decrease, followed by an increase to higher values at the outer cylinder. A localized shear stress pocket was detected in the proximity of an inner cylinder, as seen in Fig. 8 f, which corresponds to a similar phenomenon previously reported in the preceding section. Nevertheless, the stress and displacement patterns exhibit a harmonic pattern consistently across the cylinder, as seen in Fig. 2, Fig. 3, Fig. 4, Fig. 5, Fig. 6, Fig. 7, Fig. 8.

**Fig. 9:**
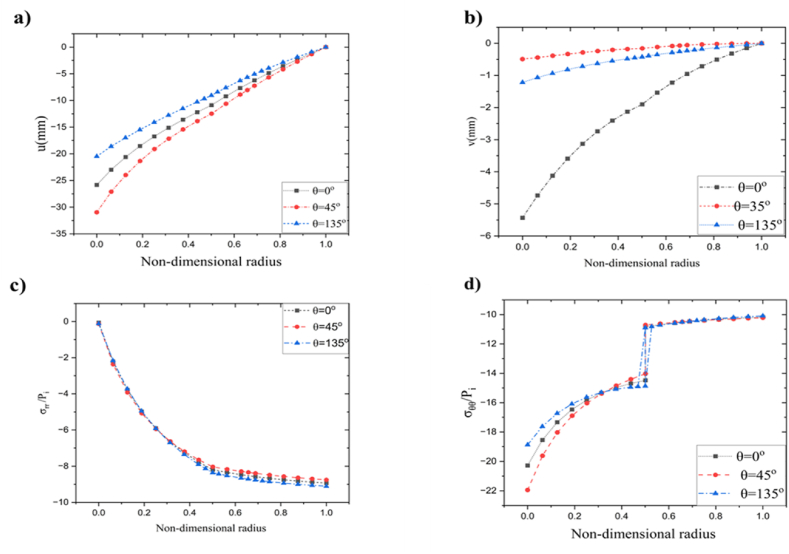

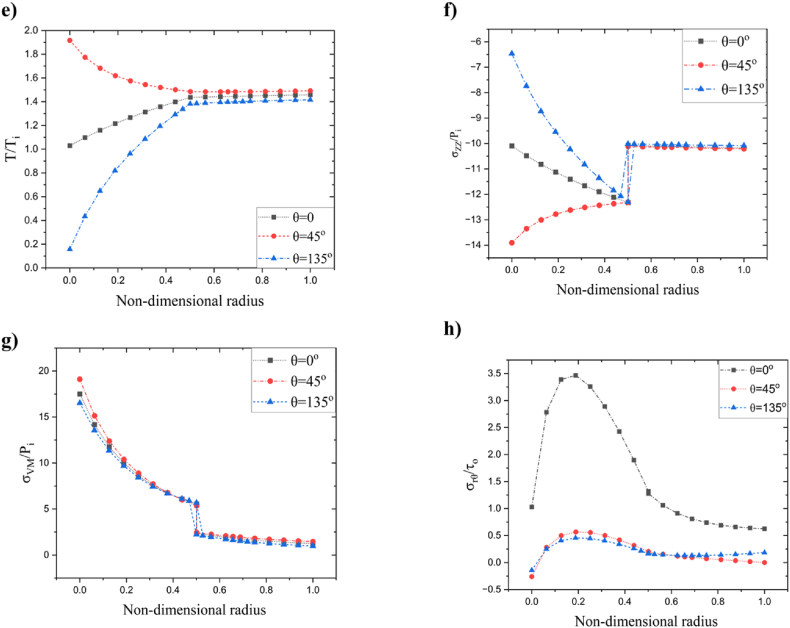
Resulting output fields at radial direction under various values of θ: a) radial displacement (u), b) tangential displacement (v), c) dimensionless radial stress, d) dimensionless hoop stress, e) dimensionless temperature, f) dimensionless axial stress, g) dimensionless failure stress, and h) dimensionless shear stress.

Additionally, Fig. 9 shows the visualization of several dependent variables across various values θ in the radial direction that fulfill the contour plot Fig. 2, Fig. 3, Fig. 4, Fig. 5, Fig. 6, Fig. 7.

However, the present suggested research has the limitation of being time-independent, which implies that nothing evolves with time. This mathematical technique is only applicable to linear and periodic B.C.s; it does not apply to aperiodic or nonlinear B.C.s, such as the influence of radiation. In addition, it cannot be applied to an unsteady loaded short cylinder with an end effect. Another potential drawback is the fact that it can only be used in regions with elastic properties.

## 6 Conclusion

This study presents an analytical and numerical solution for the non-axisymmetric thermal and mechanical stresses and displacements in a compound cylinder composed of each isotropic material. The analysis of the findings revealed that the inclusion of a 2D compound material had a positive impact on stress reduction within the cylinders, hence increasing the safety threshold for load limits. The analysis conducted in this study yields the following findings, which in turn lead to the following conclusions:

• The influence of tangential stress on the behavior of cylinders is significant and plays a dominant role.
• The augmentation of the characteristics in the outer layer has effectively amplified the radial displacement while concurrently diminishing the tangential displacement.
• Temperature changes have a greater impact on hoop stress than internal pressure levels.
• The impact of internal pressure is somewhat less pronounced when the pressure vessel is simulated using a dissimilar material.
• Displacement fields are less important in pressure vessels composed of dissimilar materials as global stiffness becomes stiffer.
• The significance of displacement fields becomes less important in the context of pressure vessels comprised of different materials due to the increased stiffness of the overall structure.
• The suggested method allows for the estimation of the behavior of the intricate pressure vessel made of functionally graded material (FGM) by employing a developed multilayered approach.

## Data availability

Data will be made available on request.

## CRediT authorship contribution statement

**Palash Das:** Writing – original draft, Methodology, Formal analysis, Data curation, Conceptualization. **Md Ashraful Islam:** Writing – review & editing, Writing – original draft, Supervision, Software, Resources, Investigation, Conceptualization. **Dipayan Mondal:** Writing – review & editing, Software, Resources.

## Declaration of competing interest

The authors declare that they have no known competing financial interests or personal relationships that could have appeared to influence the work reported in this paper.

## Figures and Tables

**Table:** 

$\tau_{11}=\frac{{r_{m}}^{2}}{h^{2}}+\frac{1}{2}\frac{r_{m}}{h}$	$\tau_{15}=\frac{1}{4}\frac{\beta_{\nu ,1}r_{m}}{hk}$	$\delta_{\nu ,1}=\left(\frac{1+\nu^{i}}{1-\nu^{i}}\right)$
$\tau_{12}=-\left(1+\frac{{r_{m}}^{2}}{h^{2}}+\frac{\alpha_{\nu ,1}}{k^{2}}\right)$	$\tau_{16}=\frac{1}{2}\frac{\gamma_{\nu ,1}}{k}$	$\alpha_{\nu ,1}=\left(\frac{1-2\nu^{i}}{2-2\nu^{i}}\right)$
$\tau_{13}=\frac{{r_{m}}^{2}}{h^{2}}-\frac{1}{2}\frac{r_{m}}{h}$	$\tau_{17}=\frac{1}{2}\frac{\delta_{\nu ,1}\alpha^{i}r_{m}^{2}\;}{h}$	$\gamma_{\nu ,1}=2\left(\frac{\nu^{i}-3}{1-\nu^{i}}\right)$
$\tau_{14}=\frac{\alpha_{\nu ,1}}{k^{2}}$	$\beta_{\nu ,1}=\left(\frac{1}{2-2\nu^{i}}\right)$	$\delta_{\nu ,2}=\left(\frac{2+2\nu^{i}}{1-2\nu^{i}}\right)$
$\tau_{21}=\tau_{12}$	$\tau_{25}=\frac{1}{4}\frac{\beta_{\nu ,2}r_{m}}{hk}$	$\alpha_{\nu ,2}=\left(\frac{2-2\nu^{i}}{1-2\nu^{i}}\right)$
$\tau_{22}=-\left(1+\frac{{r_{m}}^{2}}{h^{2}}+\frac{\alpha_{\nu ,2}}{k^{2}}\right)$	$\tau_{26}=\frac{1}{2}\frac{\gamma_{\nu ,2}}{k}$	$\gamma_{\nu ,2}=\left(\frac{3-4\nu^{i}}{1-2\nu^{i}}\right)$
$\tau_{23}=\tau_{13}$	$\tau_{27}=\frac{1}{2}\frac{\delta_{\nu ,2}\alpha^{i}r_{m}^{2}\;}{h}$	
$\tau_{24}=\frac{\alpha_{\nu ,2}}{k^{2}}$	$\beta_{\nu ,2}=\left(\frac{1}{1-2\nu^{i}}\right)$	

i=1,m=1	i=2,m=N
$\Omega_{11}=\frac{3}{2}\frac{\lambda^{i}+2\mu^{i}}{h}-\frac{\lambda^{i}}{r_{m}}$	$\Omega_{21}=-\frac{1}{2}\frac{\left(\lambda^{i}+2\mu^{i}\right)}{h}$
$\Omega_{12}=\frac{2\left(\lambda^{i}+2\mu^{i}\right)}{h}$	$\Omega_{22}=\frac{2\left(\lambda^{i}+2\mu^{i}\right)}{h}$
$\Omega_{13}=\frac{\left(\lambda^{i}+2\mu^{i}\right)}{2h}$	$\Omega_{23}=\frac{3}{2}\frac{\lambda^{i}+2\mu^{i}}{h}+\frac{\lambda^{i}}{r_{m}}$
$\Omega_{14}=\frac{\lambda^{i}}{2hr_{m}k}$	$\Omega_{24}=\frac{\lambda^{i}}{2hr_{m}k}$
$\Omega_{15}=\left(3\lambda^{i}+2\mu^{i}\right)\alpha^{i}T_{m,n}$	$\Omega_{25}=\left(3\lambda^{i}+2\mu^{i}\right)\alpha^{i}T_{m,n}$

i=1,m=l (contract surface)	i=2,m=l (contract surface)
$\Theta_{11}=\frac{1}{2}\frac{\left(\lambda^{i=1}+2\mu^{i=1}\right)}{h}$	$\Theta_{21}=-\frac{1}{2}\frac{\left(\lambda^{i=2}+2\mu^{i=2}\right)}{h}$
$\Theta_{12}=\frac{2\left(\lambda^{i=1}+2\mu^{i=1}\right)}{h}$	$\Theta_{22}=\frac{2\left(\lambda^{i=2}+2\mu^{i=2}\right)}{h}$
$\Theta_{13}=\frac{3\left(\lambda^{i=1}+2\mu^{i=1}\right)}{2h}+\frac{\lambda^{i=1}}{r_{m}}$	$\Theta_{23}=\frac{3\left(\lambda^{i=2}+2\mu^{i=2}\right)}{2h}-\frac{\lambda^{i=2}}{r_{m}}$
$\Theta_{14}=\frac{1}{2}\frac{\lambda^{i=1}}{r_{m}k}$	$\Theta_{24}=\frac{1}{2}\frac{\lambda^{i=2}}{r_{m}k}$
$\Theta_{15}=\left(3\lambda^{i=1}+2\mu^{i=1}\right)\alpha^{i=1}$	$\Theta_{25}=\left(3\lambda^{i=2}+2\mu^{i=2}\right)\alpha^{i=2}$
$\iota_{11}=\frac{\mu^{i=1}}{k}$	$\iota_{21}=\frac{\mu^{i=2}}{k}$
$\iota_{12}=\frac{\mu^{i=1}}{h}$	$\iota_{22}=\frac{\mu^{i=2}}{h}$
$\iota_{13}=\frac{4\mu^{i=1}}{h}$	$\iota_{23}=\frac{4\mu^{i=2}}{h}$
$\iota_{14}=\frac{3}{2h}+\frac{1}{r_{m}}$	$\iota_{24}=\frac{3}{2h}-\frac{1}{r_{m}}$

## References

[1] Bahoum K., Diany M., Mabrouki M. (2017). Stress analysis of compound cylinders subjected to thermo-mechanical loads. J. Mech. Sci. Technol..

[2] Clapeyron B., Lamé G. (1831).

[3] Focke E. (2007).

[4] Arefi M., Moghaddam S.K., Bidgoli E.M.-R., Kiani M., Civalek O. (2021). Analysis of graphene nanoplatelet reinforced cylindrical shell subjected to thermo-mechanical loads. Compos. Struct..

[5] Zhao W., Guo D., Gong X., Li C. (2023). Nonlinear axisymmetric buckling analysis of the FGM sandwich shallow spherical shells under thermomechanical loads. Eur. J. Mech. Solid..

[6] Benchallal R., Benslimane A., Bidgoli O., Hammiche D. (2022). Analytical solution for rotating cylindrical FGM vessel subjected to thermomechanical loadings. Mater. Today: Proc..

[7] Das P., Islam M.A., Somadder S., Hasib M.A. (2022). Analytical and numerical analysis of functionally graded (FGM) axisymmetric cylinders under thermo-mechanical loadings. Mater. Today Commun..

[8] Das P., Islam M., Somadder S., Hasib M. (2023). Analytical and numerical solutions of pressurized thick-walled FGM spheres. Arch. Appl. Mech..

[9] Benslimane A., Medjdoub C., Methia M., Khadimallah M.A., Hammiche D. (2021). Investigation of displacement and stress fields in pressurized thick-walled FGM cylinder under uniform magnetic field. Mater. Today: Proc..

[10] Shojaeifard M., Dolatabadi R., Sheikhi S., Baghani M. (2021). Coupled thermo-mechanical swelling of a thermo-responsive hydrogel hollow cylinder under extension-torsion: analytical Solution and FEM. J. Intell. Mater. Syst. Struct..

[11] Bai B., Zhou R., Cai G., Hu W., Yang G. (2021). Coupled thermo-hydro-mechanical mechanism in view of the soil particle rearrangement of granular thermodynamics. Comput. Geotech..

[12] Zhu Q., Chen J., Gou G., Chen H., Li P. (2017). Ameliorated longitudinal critically refracted—attenuation velocity method for welding residual stress measurement. J. Mater. Process. Technol..

[13] Hao R.-B., Lu Z.-Q., Ding H., Chen L.-Q. (2022). Orthogonal six-DOFs vibration isolation with tunable high-static-low-dynamic stiffness: Experiment and analysis. Int. J. Mech. Sci..

[14] Wang J., Liang F., Zhou H., Yang M., Wang Q. (2022). Analysis of Position, pose and force decoupling characteristics of a 4-UPS/1-RPS parallel grinding robot. Symmetry.

[15] Tian L.-m., Li M.-h., Li L., Li D.-y., Bai C. (2023). Novel joint for improving the collapse resistance of steel frame structures in column-loss scenarios. Thin-Walled Struct..

[16] Shi J., Zhao B., He T., Tu L., Lu X., Xu H. (2023). Tribology and dynamic characteristics of textured journal-thrust coupled bearing considering thermal and pressure coupled effects. Tribol. Int..

[17] Zhu Z., Liu Y., Gou G., Gao W., Chen J. (2021). Effect of heat input on interfacial characterization of the butter joint of hot-rolling CP-Ti/Q235 bimetallic sheets by Laser+ CMT. Sci. Rep..

[18] Cao M., Cui T., Yue Y., Li C., Guo X., Jia X., Wang B. (2023). Preparation and characterization for the thermal stability and mechanical property of PLA and PLA/CF samples built by FFF approach. Materials.

[19] Bai X., Shi H., Zhang K., Zhang X., Wu Y. (2022). Effect of the fit clearance between ceramic outer ring and steel pedestal on the sound radiation of full ceramic ball bearing system. J. Sound Vib..

[20] Bai X., Zhang Z., Shi H., Luo Z., Li T. (2023). Identification of subsurface mesoscale crack in full ceramic ball bearings based on strain energy theory. Appl. Sci..

[21] Cheng Z., Guo Z., Fu P., Yang J., Wang Q. (2021). New insights into the effects of methane and oxygen on heat/mass transfer in reactive porous media. Int. Commun. Heat Mass Tran..

[22] Baghlani A., Khayat M., Dehghan S.M. (2020). Free vibration analysis of FGM cylindrical shells surrounded by Pasternak elastic foundation in thermal environment considering fluid-structure interaction. Appl. Math. Model..

[23] Nejad M.Z., Alamzadeh N., Hadi A. (2018). Thermoelastoplastic analysis of FGM rotating thick cylindrical pressure vessels in linear elastic-fully plastic condition. Compos. B Eng..

[24] Eldeeb A., Shabana Y., Elsawaf A. (2021). Influences of angular deceleration on the thermoelastoplastic behaviors of nonuniform thickness multilayer FGM discs. Compos. Struct..

[25] Akbari A. (2021). Analytical solution of elastic–plastic stress for double-layer FGM spherical shell subjected to pressure and temperature load. J. Braz. Soc. Mech. Sci. Eng..

[26] Eldeeb A., Shabana Y., Elsawaf A. (2021). Investigation of the thermoelastoplastic behaviors of multilayer FGM cylinders. Compos. Struct..

[27] Arslan E., Mack W., Apatay T. (2021). Thermo-mechanically loaded steel/aluminum functionally graded spherical containers and pressure vessels. Int. J. Pres. Ves. Pip..

[28] Sim L., Yeo W., Purbolaksono J., Saw L., Tey J. (2021). Analytical solution of thermo-mechanical stresses of multilayered hollow spherical pressure vessel. Int. J. Pres. Ves. Pip..

[29] Babaee A., Jelovica J. (2021). Nonlinear transient thermoelastic response of FGM plate under sudden cryogenic cooling. Ocean Eng..

[30] Parker A.P. (2001). Bauschinger effect design procedures for compound tubes containing an autofrettaged layer. J. Pressure Vessel Technol..

[31] Majzoobi G., Farrahi G., Pipelzadeh M., Akbari K. (2004). Experimental and finite element prediction of bursting pressure in compound cylinders. Int. J. Pres. Ves. Pip..

[32] Parker A.P., Kendall D.P. (2003). Residual stresses and lifetimes of tubes subjected to shrink fit prior to autofrettage. J. Pressure Vessel Technol..

[33] H. Jahed, B. Farshi, and M. Karimi, "Optimum Design of Multilayered Vessels." pp. 207-213.

[34] Jahed H., Farshi B., Karimi M. (2006).

[35] M. Mohammadi, G. Farrahi, and S. Hoseini, "Determination of Residual Stresses in Autofrettaged Compound Tubes for Different Geometries." pp. 53-61.

[36] Jabbari M., Sohrabpour S., Eslami M. (2003). General solution for mechanical and thermal stresses in a functionally graded hollow cylinder due to nonaxisymmetric steady-state loads. J. Appl. Mech..

[37] Jabbari M., Bahtui A., Eslami M. (2009). Axisymmetric mechanical and thermal stresses in thick short length FGM cylinders. Int. J. Pres. Ves. Pip..

[38] Poultangari R., Jabbari M., Eslami M. (2008). Functionally graded hollow spheres under non-axisymmetric thermo-mechanical loads. Int. J. Pres. Ves. Pip..

[39] Nohut S., Schwentenwein M. (2022). Vat photopolymerization additive manufacturing of functionally graded materials: a review. Journal of Manufacturing and Materials Processing.

[40] Loghman A., Nasr M., Arefi M. (2017). Nonsymmetric thermomechanical analysis of a functionally graded cylinder subjected to mechanical, thermal, and magnetic loads. J. Therm. Stresses.

[41] Eslami M.R., Hetnarski R.B., Ignaczak J., Noda N., Sumi N., Tanigawa Y. (2013).

[42] Lurie A.I. (2010).

[43] Wang X., Wang Z., Zeng T., Cheng S., Yang F. (2018). Exact analytical solution for steady-state heat transfer in functionally graded sandwich slabs with convective-radiative boundary conditions. Compos. Struct..

